# Fluorescence imaging of MALAT1 expression using a Cy5.5-labeled antisense oligonucleotide in lung cancer and epidermal carcinoma cells

**DOI:** 10.1186/s40644-025-00903-y

**Published:** 2025-07-01

**Authors:** Zhenfeng Liu, Chengjun Yao, Haopeng Ni, Guolin Wang, Mengjie Dong

**Affiliations:** 1 Department of Nuclear Medicine, The First Affiliated Hospital, School of Medicine, Zhejiang University, #79 Qingchun Road, Hangzhou, 310003 Zhejiang Province China; 2 Zhejiang University School of Medicine, #866 Yuhangtang Road, Hangzhou, 3100058 Zhejiang Province China

**Keywords:** Metastasis associated lung adenocarcinoma transcript 1, Antisense oligonucleotides, Cyanine5.5, Optical fluorescence imaging, Pan-cancer research

## Abstract

**Background:**

The long noncoding RNA Metastasis Associated Lung Adenocarcinoma Transcript 1 (MALAT1) has been extensively studied as an oncogenic factor. Antisense oligonucleotides (ASOs) labeled with the Cyanine5.5 (Cy5.5) dye enable effective in vivo imaging using near-infrared fluorescence.

**Methods:**

Pan-cancer research on MALAT1 expression levels was conducted through The Cancer Genome Atlas (TCGA) database analysis. The selectivity and specificity of MALAT1-ASO were validated in lung cancer and epidermal carcinoma cell lines (A549, A431, PC9GR, and PC9) using cellular fluorescence and flow cytometry. Corresponding xenograft models were created for these cell lines, and near-infrared fluorescence imaging assessed tumor imaging effectiveness and the biodistribution of Cy5.5-labeled MALAT1 ASOs.

**Results:**

MALAT1 expression levels were found to be upregulated in various tumors and high MALAT1 expression level correlated to poor prognosis in some tumors. The high expression of MALAT1 was confirmed in tumor cell lines. In vitro fluorescent intensity correlated with MALAT1 expression within cells. The fluorescence intensity also exhibited concentration dependence. In vivo experiments revealed a significant contrast between tumor tissues and normal tissues within 24 h. Tumors exhibited varied probe uptake corresponding to their MALAT1 expression levels. Ex vivo experiments shows high probe uptake in kidney, liver and intestine tissues.

**Conclusion:**

MALAT1 is highly expressed in various cancer tissues and associated with poor prognosis. In xenograft models of lung cancer and epidermal carcinoma cell lines A549, A431, PC9GR, and PC9, Cy5.5-labeled ASOs exhibit evident binding specificity and discernible imaging effect in both in vitro and in vivo, effectively reflecting MALAT1 expression levels in tumors.

**Supplementary Information:**

The online version contains supplementary material available at 10.1186/s40644-025-00903-y.

## Introduction

Long non-coding RNAs (lncRNAs) are transcribed RNA molecules that are greater than 200 nucleotides in length [1]. Despite not being translated into proteins, they play a pivotal role in regulating various aspects of tumor proliferation, migration, invasion, and apoptosis through diverse mechanisms. LncRNAs can modulate epigenetic gene regulation, such as HOX transcript antisense RNA (HOTAIR) and X-inactive specific transcript (Xist), which have been found to interact with the Polycomb repressive complex 2 (PRC2), thereby inactivating chromatin through the establishment of inhibitory H3K427me3 histone marks [2]. Additionally, lncRNA-tumor necrosis factor alpha-induced protein 3 (TNFAIP3) can form a complex with high mobility group box 1 (HMGB1), resulting in the regulation of HMGB1-related histone modifications [3]. Furthermore, lncRNAs can interact with proteins, for instance, C-terminal binding protein 1 antisense RNA (CTBP1-AS) and colon cancer–associated transcript 2 (CCTA2), which interact with transcription factors and modify their activity [4]. Moreover, lncRNAs can function as competing endogenous RNAs, participating in transcriptional and post-transcriptional regulation of genes [5]. LncRNAs are involved throughout the entire process of tumor growth. For example plasmacytoma variant translocation 1 (PVT1), which can affect the proliferation of thyroid cancer by regulating the abundance of the thyroid-stimulating hormone receptor [6]. HOTAIR can promote angiogenesis by directly activating the transcription of the angiogenic factor VEGFA [7]. LncRNAs like H19, MALAT1, sprouty4 intronic transcript 1 (SPRY4-IT1), and actin filament associated protein 1 antisense RNA 1 (AFAP1-AS1) have all been found to be associated with metastasis in various types of cancer [8, 9].

Due to the crucial roles of lncRNAs, recent research has explored their potential as biomarkers for cancer diagnosis [10]For instance, prostate cancer antigen 3 (PCA3) in urine samples has been approved by the US Food and Drug Administration as a biomarker for prostate cancer [11]. H19 has demonstrated its effectiveness in the early diagnosis of gastric cancer with a sensitivity of 85.5% and a specificity of 80.1% [12]. Meanwhile, MALAT1, which differentially expressed in multiple cancer types, has garnered attention as a potential biomarker in recent studies [13].

MALAT1, also recognized as Nuclear Enriched Abundant Transcript 2 (NEAT2), is situated on human chromosome 11q13.12 [14]. Initially discovered in the context of non-small cell lung cancer (NSCLC) research [15]MALAT1’s overexpression is linked not only to lung cancer but also to tumors with heightened metastatic potential [9, 15–17]. Moreover, MALAT1 is implicated in the progression of various malignant tumors beyond lung cancer [18]. Huang et al. found that high MALAT1 expression was associated with positive estrogen receptor status in breast cancer, and patients with high MALAT1 expression had poorer survival curves [17]. Yang et al. discovered that MALAT1 promotes colorectal cancer cell proliferation, migration, and invasion by upregulating PRKA kinase anchor protein 9 (AKAP-9) [9]. Liu et al. reviewed the pro-oncogenic mechanisms of MALAT1, including mediating alternative splicing, regulating epigenetics by modulating PRC2, and participating in a reciprocal interaction with microRNAs. Additionally, they highlighted several signaling pathways, such as Wnt/β-catenin, PI3K/AKT, ERK/MAPK, and angiogenesis [19].

Lung cancer stands as the foremost cause of cancer-related deaths globally [20]. Following malignant melanoma, epidermal carcinoma holds the second position in mortality rates among skin tumors. Both of these cancers exhibit high malignancy and are capable of metastasis. While conventional imaging and pathological diagnostics enable accurate diagnosis and staging of these tumors, the development of targeted therapies has emphasized the importance of genetic and molecular level assessments. Notably, changes in specific molecular markers in some tumors can render targeted therapies ineffective, such as gefitinib resistance in lung cancer, which further underscores the need for molecular imaging. With its high expression in various tumor tissues and clinical relevance, MALAT1 emerges as a promising target for developing a molecular imaging diagnostic method.

Currently, various imaging methods are widely used for clinical tumor observations, including magnetic resonance imaging (MRI), X-ray computed tomography (CT), single photon emission computed tomography (SPECT), positron emission tomography (PET), ultrasound (US), and more. Optical imaging (OI), as a novel molecular imaging technology, offers significant potential applications and research opportunities in clinical tumor diagnosis and treatment. OI is a technique that employs optical detection methods to image cells, tissues, and even whole organisms by utilizing fluorescence effects within the visible and near-infrared light spectrum for real-time quantitative analysis. The advantages of OI include non-invasiveness, the ability for continuous and repeatable detection, rapid real-time scanning and imaging, high sensitivity, and minimal adverse reactions. Differing from the contrast agents commonly used with CT and MRI, which may impair renal function [21]fluorescence imaging probes exhibit high sensitivity and can be detected at very low concentrations, thereby reducing the risk of toxicity to patients [22]. In comparison with PET, OI does not involve radioactive isotopes, thus eliminating radiation exposure and making it safer for long-term and repeated imaging. Furthermore, OI systems are generally more cost-effective and accessible. Cy5.5 dye can be combined with various proteins, antibodies and nucleic acids to create targeted imaging probes. Over the past two decades, relative researches had repeatedly confirmed advantages and safety of Cy5.5 dye as a real-time in vivo optical imaging dye [23]. Fluorescence imaging has been applied in various medical contexts, such as tumor angiography, lymphatic imaging [24] and thyroid surgery [25] to avoid accidental parathyroid gland resection.

The development of molecular probes for tumor-specific optical imaging holds significant importance [26]. One approach to designing probes for lncRNA involves synthesizing corresponding antisense oligonucleotides (ASOs). ASOs are artificially synthesized oligonucleotide fragments of DNA or RNA, comprising 13–25 nucleotides, capable of binding to target genes or target RNA through base complementarity rules [27, 28]. ASOs have strong penetrability, lack of immunogenicity, and high specificity for binding to target molecules [27]. Therefore, after fluorescence and radioisotope labeling, they can serve as molecular probes in tumor diagnosis and the genetic-level functional evaluation of tumors.

Our team has previously utilized 68Ga-labeled MALAT1-ASOs for PET/CT imaging, achieving promising results in liver cancer LM3 cell lines and xenograft models [29]. Furthermore, we innovatively employed Cy5.5-labeled MALAT1-ASOs for near-infrared spectral imaging in LM3^30^. While Cy5.5-labeled MALAT1-ASOs demonstrated excellent imaging effects in liver cancer, their potential application in other tumors requires further validation. In this study, we initially investigated the MALAT1 expression across various tumors and explored its correlation with tumor prognosis through comprehensive database analysis. Additionally, we validated the imaging efficacy of Cy5.5-labeled MALAT1-ASO probes using lung cancer and skin squamous cell carcinoma-related cell lines in both in vitro and in vivo experiments. Through dynamic fluorescence imaging in live animals using this probe, we can assess the MALAT1 expression levels in different tumors, thereby providing valuable data for tumor treatment.

## Materials and methods

### General

All chemicals, were purchased from commercial suppliers and used without further purification. Commonly used cell culture reagents, including DMEM, fetal bovine serum (FBS), PBS, and Tyrisin, were purchased from Sigma-Aldrich. The usage protocols were in accordance with the manufacturer’s instructions and were consistent with those described in a previous article [30]. For other specific experimental reagents, their respective manufacturers are mentioned later in this document, and they were strictly used following the provided instructions. The probes MALAT1-ASO (MALAT1-specific antisense oligonucleotides), 6-FAM-MALAT1-ASO (6-Carboxyfluorescein labeled MALAT1-ASO) and Cy5.5-MALAT1-ASO (Cyanine5.5 labeled MALAT1-ASO) were designed by us with the base sequence used was follows: GGGAGTTACTTGCCAACTTG (Figure S1). The synthesized probes were purchased from Sangon Biotech, inc., Shanghai, China. The properties of the synthesized compounds and the results of mass spectrometry analysis were provided by the company, and we have obtained the copyright to utilize this data.

### Pan-cancer MALAT1 expression analysis

The gene ENSG00000279576 (MALAT1) expression was examined using the Tumor Immune Estimation Resource version 2 (Timer2; http://timer.cistrome.org/), a tool that can access TCGA data to compare expression levels across various tumors and adjacent normal tissues. Additionally, Gene Expression Profiling Interactive Analysis version 2 (GEPIA2; http://gepia2.cancer-pku.cn/) was employed to analyze MALAT1 expression levels by using TCGA and Genotype-Tissue Expression Project (GTEx) normal tissue database through the “Expression DIY-Box Plots” function. MALAT1 expression in different tumors based on staging was assessed using the “Expression DIY-Stage Plots” function of GEPIA2. Significance analysis for all data was performed using non-paired Wilcoxon Rank Sum and Signed Rank Tests.

### Pan-cancer MALAT1 survival prognosis analysis

The correlation between survival prognosis and MALAT1 was explored across different tumors. The “Survival analysis” function in GEPIA2 was used to analyze overall survival (OS) and disease-free survival (DFS) data across all TCGA tumors. High and low MALAT1 expression groups were differentiated with a 50% cut-off. Significance analysis was conducted using Log-rank analysis, and corresponding Kaplan-Meier survival plots were generated for all data.

### Absorption and emission spectra

The absorption spectrum of labeled probes was recorded on a UV–Vis spectrometer (UV-2600, Shimadzu) and the scanning range was from 350 to 600 nm for 6-FAM-MALAT1-ASO, and 550 to 800 nm for Cy5.5-MALAT1 ASO with an increment of 0.1 nm. The fluorescence emission of labeled probes was then measured with a spectrofluorophotometer (RF-6000, Shimadzu) and the spectrum was scanned from 350 to 600 nm for 6-FAM-MALAT1-ASO, and 550 to 800 nm for Cy5.5-MALAT1 ASO with an increment of 0.1 nm. The wave length of the excitation light for 6-FAM-MALAT1-ASO and Cy5.5-MALAT1 ASO was set at 450 nm and 620 nm respectively.

### In vitro stability analysis

In vitro stability studies of Cy5.5-MALAT1-ASO were performed in phosphate buffer solution (PBS, sigma) and fetal bovine serum (FBS, sigma) solutions. Briefly, 5 nmol of MALAT1 ASO was incubated in PBS or fresh 37 ° C fetal bovine serum for 2 h, 24 h, and 48 h. Then a certain amount of Cy5.5-MALAT1-ASO in PBS solution was taken, and its purity was analyzed by HPLC to monitor its stability. For stability analysis in serum, serum proteins were precipitated by the addition of 0.5 mL acetonitrile, and the solution was then centrifuged, the stability of the supernatant in serum was also determined by HPLC (UV,250 nm).

### Cell culture

Human umbilical vein endothelial cells (HUVEC), human lung adenocarcinoma cells (A549), and human epidermal carcinoma cells (A431) were graciously provided by Prof. Ben Wang’s laboratory, Institution of Translational Medicine, Zhejiang University. The human lung cancer cells (PC9) and the gefitinib-resistant strain of PC9 cells (PC9GR) were generously donated by the Respiratory Department Laboratory at the First Affiliated Hospital, School of Medicine, Zhejiang University. These cell lines were cultured in Dulbecco’s Modified Eagle Medium (DMEM, sigma) supplemented with 10% fetal bovine serum (FBS, sigma) with 100 U/mL penicillin and 100 µg/mL streptomycin. The cells were maintained in a humidified atmosphere of 5% CO2 at 37 °C, with the culture medium being changed every 2–3 days to sustain cell viability and proliferation.

### RNA isolation and quantitative real-time RT–PCR (q-PCR)

The specific procedures were the same as articles published previously [29, 31]. All cell lines were first separately passaged into six-well plates (Corning, USA). Total RNAs of cells in each well were extracted using EZ-press RNA Purification Kit (B0004D, EZBioscience, MN, USA) following the manufacturer’s protocol. Then, complementary DNA (cDNA) was then synthesized using EZscript Reverse Transcription Mix II (EZB-RT2GQ, EZBioscience, MN, USA). The expression of MALAT1 was determined by q-PCR using the ABI7500 system (Applied Biosystems, CA, USA) and SYBR Green qPCR Master Mix (A0001-R2, EZBioscience, Roseville, USA). The relative expression of the gene was calculated using GAPDH as an endogenous control. The primer sequences for GAPDH were 5’-GTCTCCTCTGACTTCAACAGCG-3’ (sense) and 5’-ACCACCCTGTTG-CTGTAGCCAA-3’ (antisense). The primer sequences for MALAT1 were 5’-GAATTGCGTCATTTAAAGCCTAGTT-3’ (sense) and 5’-GTTTCATCCTACCACTCCCAA-TTAAT-3’ (antisense) [32]. All samples were normalized using the 2-ΔΔCT method.

### In vitro fluorescence imaging of 6-FAM-MALAT1 ASO

Cell lines A549, PC9, PC9GR, A431, and HUVECs were added into 48-well flat-bottomed culture plates (Corning, USA) with 200µL medium (DMEM supplemented with 10%FBS) for 24 h, and the density of cells per well was 1 × 10^5^/ml. After washing with phosphate buffered saline (PBS) for 3 min, the cells in each well were fixed with 4% paraformaldehyde (PFA) for 10 min, and then washed with PBS three times (3 minutes per wash). In the fluorescence imaging experiment, for non-block group, the A549, PC9, PC9GR, A431 and HUVEC cells were incubated with 80µM of 6-FAM-MALAT1 ASO in 200µL PBS at 37℃ in the dark for 4 hours. As for block group, the A549, PC9, PC9GR, A431 and HUVEC cells were first incubated with 200µM of unlabeled MALAT1 ASO in 200µL PBS at 37℃ in the dark for 1.5 hours, followed by incubation with 80 µM of 6-FAM-MALAT1 ASO for another 4 hours. In the control group, cells were incubated with 200µL PBS for 4 hours. After incubation, the cells were washed three times with PBS (3 minutes per wash). Each well was then stained with DAPI (4’,6-diamidino-2-phenylindole, Biosharp, China) for 15 min to label the cell nuclei, followed by observation and collection of fluorescence images using the Olympus IX81 fluorescence microscope. The excitation wavelengths for 6-FAM-MALAT1 ASO and DAPI are 480 nm and 360 nm, respectively. In the concentration gradient experiment, the PC9 cells were incubated with different final concentrations (0µM, 30µM, 40µM, 80µM) of 6-FAM-MALAT1 ASO for 4 h. Subsequent procedures were the same as previously described. Confocal imaging was performed using a 24-well plate with 14 mm diameter cell slides. Staining procedures were performed as previously described. Imaging was conducted using a Leica Stellaris 5 confocal microscope equipped with a 63× objective lens. F-actin was labeled with rhodamine-conjugated phalloidin (Solarbio, Beijing, China) and co-stained with DAPI.

### Flow cytometry analysis

PC9 and HUVEC cell lines were pre-plated in 6-well plates. Once the cells reached near confluence, they were digested using EDTA-free trypsin and divided into ‘block’ and ‘non-block’ groups. The cells were fixed with 4% paraformaldehyde (PFA) for 10 min. In the non-block group, they were then incubated with 10µM 6-FAM-MALAT1 ASO for 1 h. In the block group, the cells were pre-incubated with 200µM of unlabeled MALAT1 ASO and 10µM 6-FAM-MALAT1 ASO for 1 h. After each step, the cells were washed with PBS. Finally, fluorescence intensity was measured for 10,000 cells using a flow cytometer (CytoFLEX, Beckman), and data analysis and graphing were performed using FlowJo v10.8.1 software. The average relative fluorescence intensity of the FITC channel of cells was analyzed.

### Animals and xenograft models

All animal studies were conducted in compliance with the guidelines and protocols established by the Institutional Animal Care and Use Committee of the First Affiliated Hospital of Zhejiang University School of Medicine. The ethical approvals were authorized by the Tab of Animal Experimental Ethical Inspection of the First Affiliated Hospital (Reference Number: 20201131). BALB/c nu/nu mice (female, 20 ± 2 g, 4- to 6-wk-old; Department of Laboratory Animal Science, Zhejiang University) were used in this study. Tumor cells (8 × 10^5^ cells) were subcutaneously injected into the flanks of the mice and allowed to grow for 2–4 weeks until the tumors reached a volume of 1 ± 0.5 cm [3]. HUVEC cells were used as a control to establish a similar model, but they did not form tumors. The mice were kept in a standard environment with free access to food and water, and maintained on a standard diet and bedding. All mice were euthanized in a carbon dioxide chamber at the end of the experiment.

### MALAT1 expression in nude mice organs and human organ

Various organs were harvested from A431 xenograft nude mice models (*N* = 6) post-euthanasia using a CO_2_ chamber. Total RNA was extracted from each organ and subjected to the aforementioned q-PCR method to assess MALAT1 gene expression levels. Additionally, MALAT1 expression data in human organs were sourced from the NCBI database Bioproject: PRJEB4337 (https://www.ncbi.nlm.nih.gov/bioproject/PRJEB4337/). This dataset, conducted by Fagerberg et al. in 2014, is based on a transcriptome study comprising 95 human samples across 27 different tissues [33].

### In vivo and ex vivo near-infrared fluorescence imaging

The In vivo fluorescence imaging was performed on nude mouse xenograft models of A431, A549, PC9, and PC9GR with comparison of HUVEC cells injected nude mice (with no tumor formation). The procedure is similar to previous article [30]. All mice of each tumor model were randomly assigned in to non-block group (*n* = 6) and block group (*n* = 4). The tumor-bearing nude mice in the non-block group were intravenously injected with 1.5 nmol of Cy5.5-MALAT1 ASO via the tail vein, while the mice in the block group were injected with a mixture of 200 nmol of unlabeled MALAT1 ASO and 1.5 nmol of Cy5.5-MALAT1 ASO. Then, the mice were first anesthetized with isoflurane. The near-infrared fluorescence imaging was performed at several time points (0.5 h, 1 h, 2 hours, 8 h, 24 h, 48 h) using the IVIS Imaging System 200 Series and analyzed with the IVIS Living Imaging 4.4 software (PerkinElmer Inc., Alameda, CA, USA). The fluorescence emission images were normalized and expressed as photons per second per centimeter squared per steradian (p/s/cm^2^/sr). The images were acquired with 1 s exposure time (f/stop = 4). After 48 h, the mice from the non-block and block groups were euthanized, and their tumors, tissues, and organs were dissected and subjected to ex vivo fluorescence imaging. The probe biodistribution in the HUVEC injection model at 30 min to 48 h were studied by ex vivo fluorescent imaging.

### Assessment of liver and kidney function

To assess potential hepatotoxicity and nephrotoxicity, blood samples were collected from mice after euthanasia, 72 h following a 1.5 nmol injection of Cy5.5-MALAT1 ASO. The samples were allowed to clot at room temperature and then centrifuged at 3000 × g for 10 min to obtain serum. Serum levels of alanine aminotransferase (ALT), aspartate aminotransferase (AST), blood urea nitrogen (BUN), and creatinine (CREA) were measured using an automated biochemical analyzer (Mindray, China) according to the manufacturer’s instructions. Sample number *N* = 6 and results were expressed as mean ± standard deviation (SD).

### Statistical analysis

Data analysis was conducted using SPSS 26.0 software (SPSS, IL, USA) and GraphPad Prism 9.0 (GraphPad Software, CA, USA). ImageJ (NIH, MD, USA) was employed for image processing and analysis. The copyright licenses for the usage of all software tools in this study were obtained by our institution. All quantitative data in the experiment were presented as mean ± standard deviation (SD). Normalization tests were performed on all data, and for normally distributed data, a two-tailed Student’s t-test was applied to compare differences between two groups. Analysis of variance (one-way ANOVA) was utilized for comparisons involving multiple groups. For non-normally distributed data, non-parametric testing methods were employed for comparisons. A p-value less than 0.05 was considered statistically significant.

## Result

### Pan-cancer MALAT1 expression and survival prognosis analysis

The expression levels of the MALAT1 gene were compared across various tumors in the TCGA dataset using the TIMER2 tool. As shown in Figure S2A, elevated MALAT1 expression was observed in nine tumor tissues compared to normal tissues, including Lung Adenocarcinoma (LUAD), Cholangiocarcinoma (CHOL), Colon Adenocarcinoma (COAD), Head and Neck Squamous Cell Carcinoma (HNSC), etc. We further investigated GTEx dataset for normal tissues using the GEPIA2 tool (Figure S2B), we found significant upregulation of MALAT1 expression in four tumors, such as CHOL, Esophageal Carcinoma (ESCA), Acute Myeloid Leukemia (LAML), and Stomach Adenocarcinoma (STAD). Furthermore, we analyzed MALAT1 expression in different stages of various tumors in the TCGA database (Figure S2C), revealing differences in MALAT1 expression among different stages in five tumors including LUAD. In TCGA dataset, as shown in Figure S3A, we identified a correlation between high MALAT1 expression and poorer overall survival in Kidney Renal Clear Cell Carcinoma (KIRC). Additionally, elevated MALAT1 expression was associated with a significant decrease in disease-free survival in COAD, Liver Hepatocellular Carcinoma (LIHC), and Prostate Adenocarcinoma (PRAD). The corresponding Kaplan-Meier plots (Figure S3B) demonstrated a significant reduction in median survival for high MALAT1 expression in these four tumors.

### Mass spectrometry identification

A mass spectrometry analysis was conducted for compound verification. Mass spectrometry for MALAT1-ASO、5′-6-FAM-MALAT1-ASO、5′-Cy5.5-MALAT1-ASO yielded [M-H]^−^ of 6148.4 (calculated, 6148.06)、6884.8 (calculated, 6684.66)、7072.4 (calculated, 7072.06) respectively (Figure S4A-C).

### Absorption and emission spectra

The 6-FAM-MALAT1 ASO and Cy5.5-MALAT1 ASO displayed absorption and fluorescence emission spectra as shown in Figure S4D-E. The peak of the maximum absorption and fluorescence emission for 6-FAM -MALAT1 ASO was determined to be 458 nm and 518 nm, respectively. As for Cy5.5-MALAT1 ASO, the maximum absorption and fluorescence emission was determined to be 686 nm and 706 nm, respectively.

### In vitro stability

The in vitro stability of Cy5.5-MALAT1-ASO was evaluated using HPLC. The chromatograms in PBS and FBS at 2 h, 24 h, and 48 h exhibited nearly identical profiles, indicating that the chemical purity of the probe was > 95% in PBS and > 94% in FBS at 48 h. The probe demonstrated sustained stability over 48 h, showing minimal decomposition into other by-products under simulated biological conditions (Figure S4F).

### Expression of MALAT1 in tumor cell lines

**Fig. 1:**
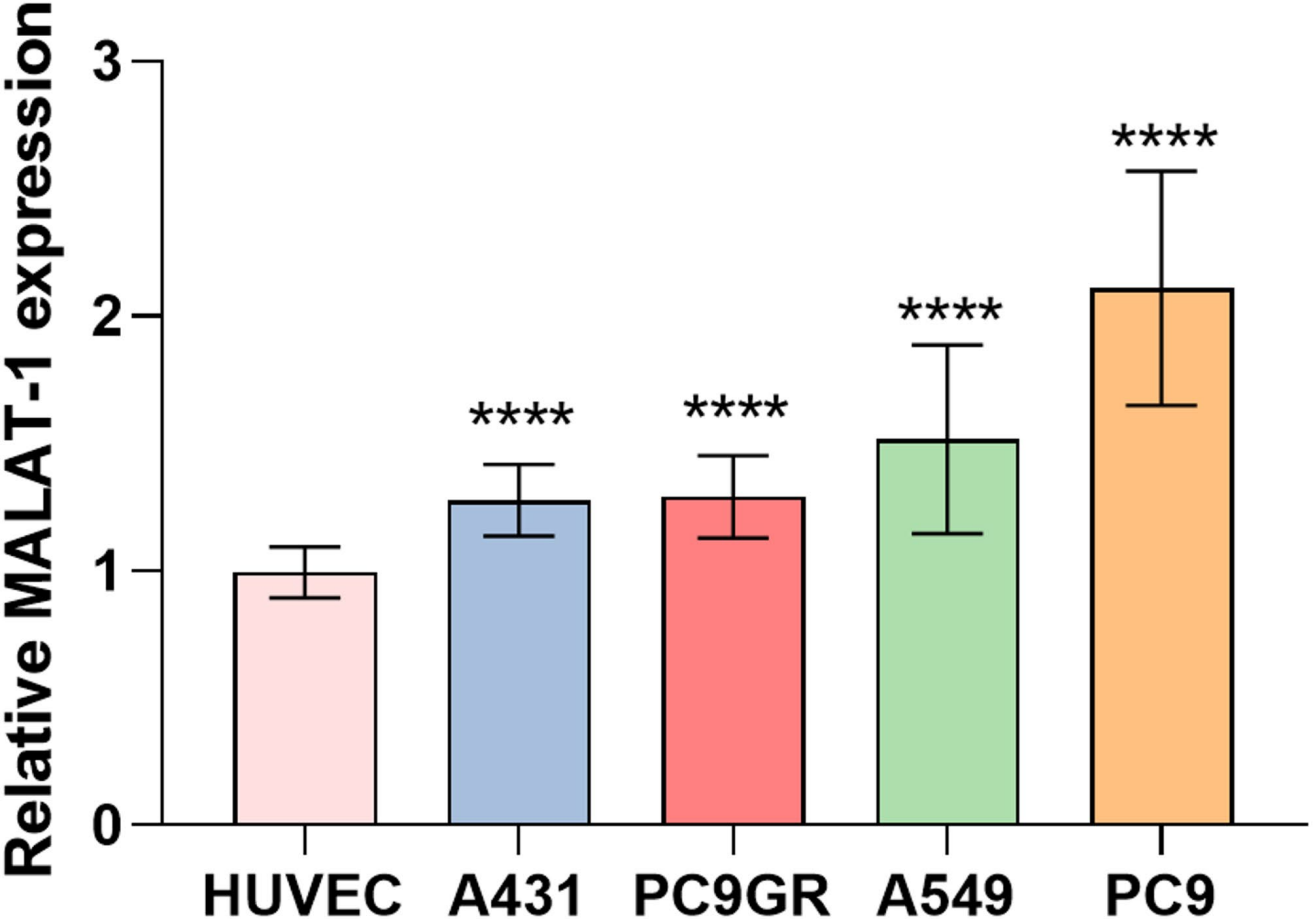
The Expression Levels of MALAT1 in Cell Lines. All tumor cell lines had significantly higher expression of MALAT1 compared to HUVEC cells. *N* = 12, ****P < 0.001, two-tailed Student’s t-test

The expression of MALAT1 in tumor cell lines (A431, A549, PC9GR, PC9) was evaluated using quantitative real-time PCR and normalized to the endogenous control GAPDH (Fig. 1). Results showed a significant increase in the expression of MALAT1 in all four tumor cell lines compared to HUVEC used as a control. The order of increased expression levels from highest to lowest was observed in PC9 (2.10 ± 0.44, *P* < 0.001), A549 (1.42 ± 0.38, *P* < 0.005), PC9GR (1.29 ± 0.15, *P* < 0.001), and A431 (1.28 ± 0.13, *P* < 0.001).

### Binding specificity of MALAT1 ASO in vitro

**Fig. 2:**
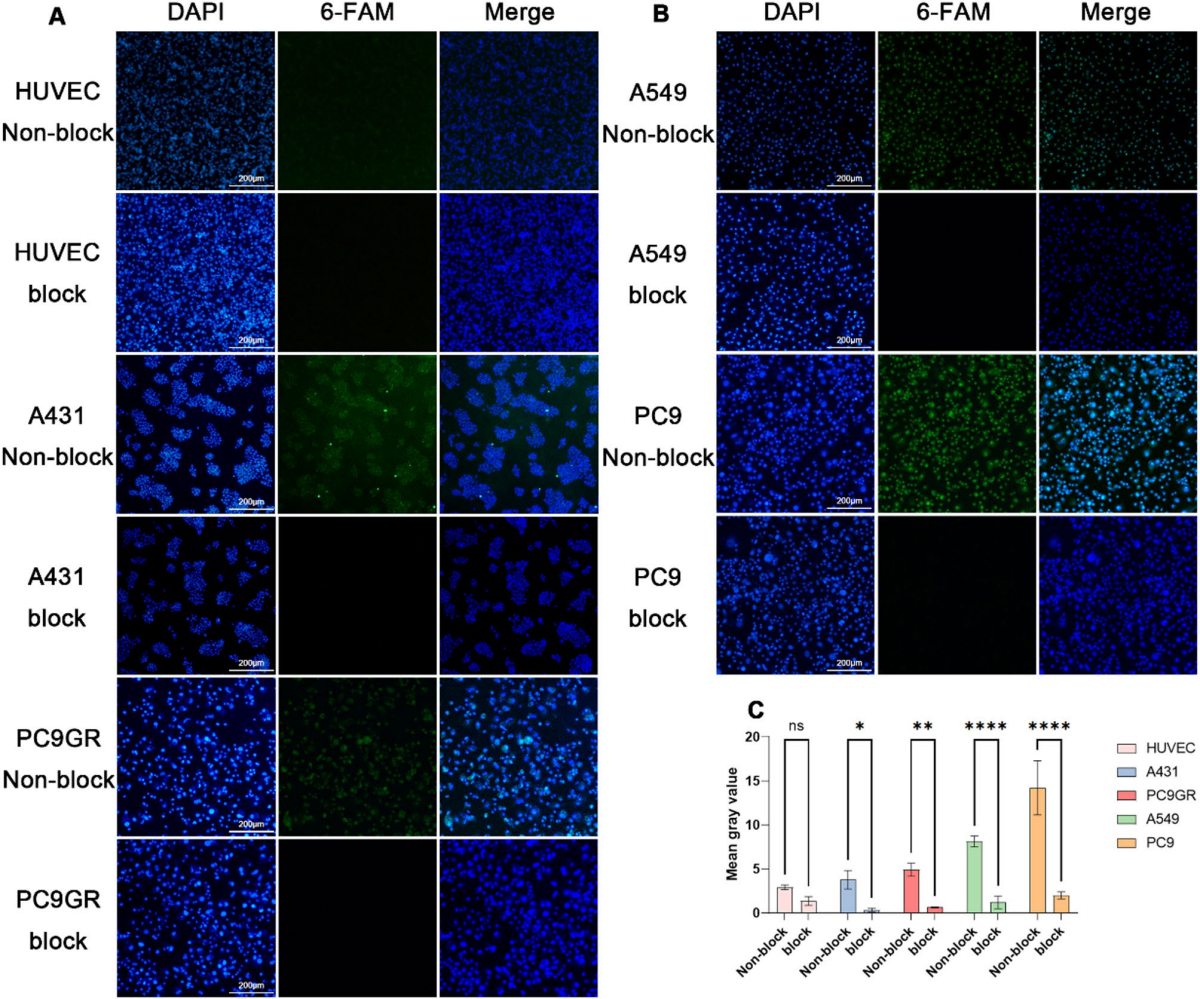
The Fluorescence Imaging of 6-FAM-MALAT1 ASO in Cell lines. Block and unblock fluorescence Imaging of (**A**) HUVEC, A431, PC9GR; (**B**) A549, PC9. In each group, the DAPI, 6-FAM and merged photos are presented. (**C**) Mean gray value analysis of fluorescence images, *P < 0.05, **P < 0.01, ****P < 0.001, two-tailed Student’s t-test

**Fig. 3:**
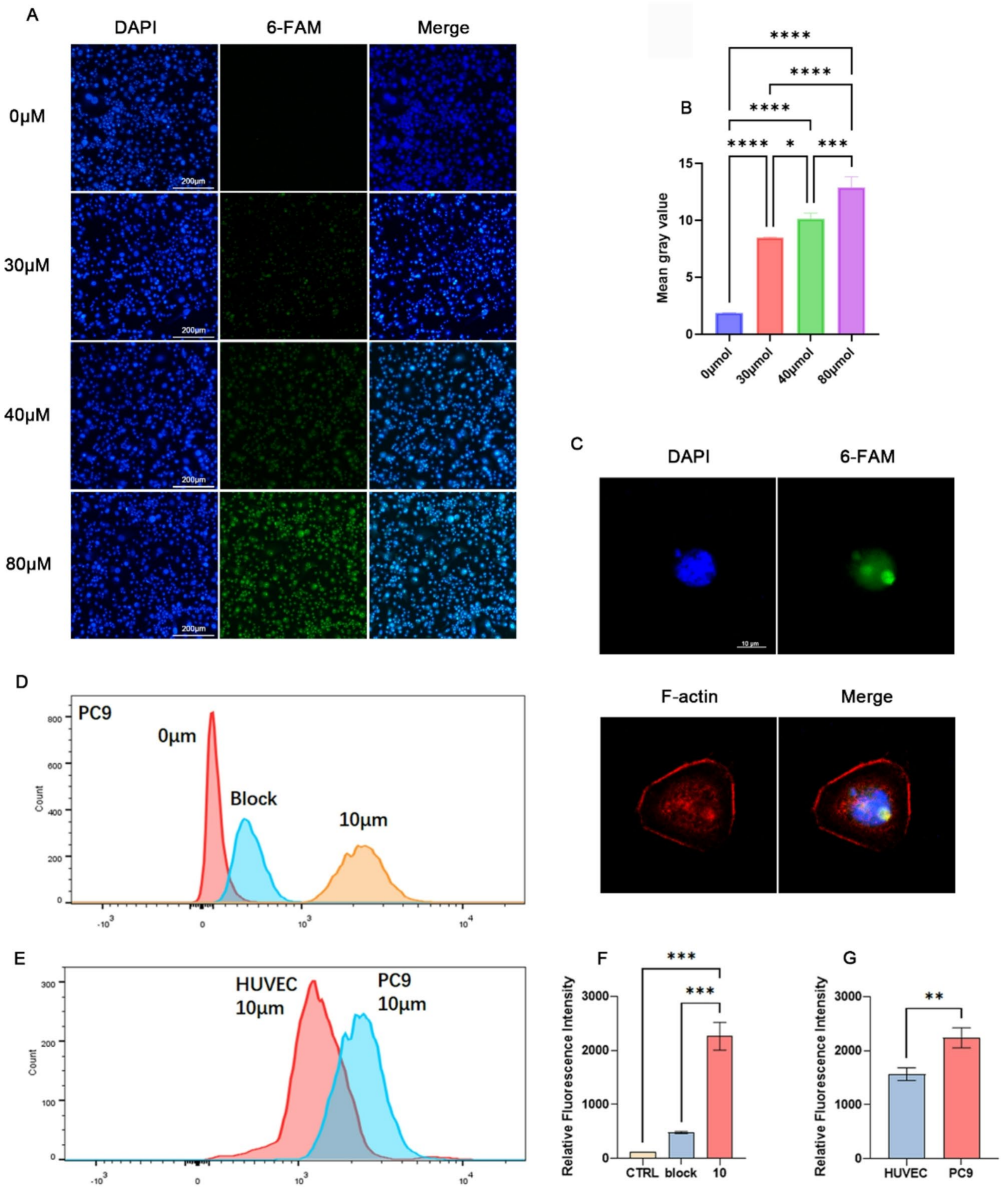
The 6-FAM-MALAT1 ASO Specifically Target and Bind to MALAT1 in vitro. (**A**) Fluorescence imagings of 6-FAM-MALAT1-ASO in PC9 with gradient of final concentration, the fluorescence signal increased with increasing 6-FAM-MALAT1 ASO concentrations. (**B**) Mean gray value analysis of gradient fluorescence images, *P < 0.05, ***P < 0.005, ****P < 0.001. (**C**) Intracellular localization of MALAT1, PC9 cells were labeled with the probe and imaged using a confocal microscope. (**D**-**E**) Flow cytometry analysis of probe binding and specific inhibition. Each group underwent three independent measurements, with a total of 10,000 cells counted per measurement. The block group was treated with 10 μm 6-FAM-MALAT1 ASO and 200 μm unlabeled MALAT1 ASO. (**F**-**G**) Statistic analysis of flow cytometry results. **P < 0.01, ***P < 0.005

6-FAM-MALAT1 ASO was assessed for specific cellular uptake through fluorescence imaging (Fig. 2A, B). Cells including HUVEC, A431, PC9GR, A549, and PC9 were incubated with the probe at a concentration of 80µM. The results showed minimal fluorescent signal of 6-FAM in HUVEC cells, whereas in A431, PC9GR, A549, and PC9 cells, the fluorescence intensity increased sequentially. In the block group, preincubation of 200µM unlabeled MALAT1 ASO significantly reduced the fluorescent signals compared to the non-block group (Fig. 2C). Furthermore, in the concentration gradient experiment, the fluorescence signal in PC9 cells increased with increasing 6-FAM-MALAT1 ASO concentrations from 0µM to 80µM. (Fig. 3A, B). In addition, confocal microscopic analysis showed that MALAT1 was mainly distributed in the nucleus and with few signals in the cytoplasm (Fig. 3C, Figure S5).

### Flow cytometry for probe binding efficiency and specific Inhibition

The binding specificity of MALAT1 ASO to PC9 and HUVEC cells was determined by flow cytometry analysis (Fig. 3D-G). The results revealed a complete separation of the fluorescence peaks between the block and unblock conditions in the PC9 cell line. Moreover, compared to HUVEC at 10 μm, PC9 cells exhibited a higher fluorescence intensity.

### Expression of MALAT1 in mouse and human tissues

**Fig. 4:**
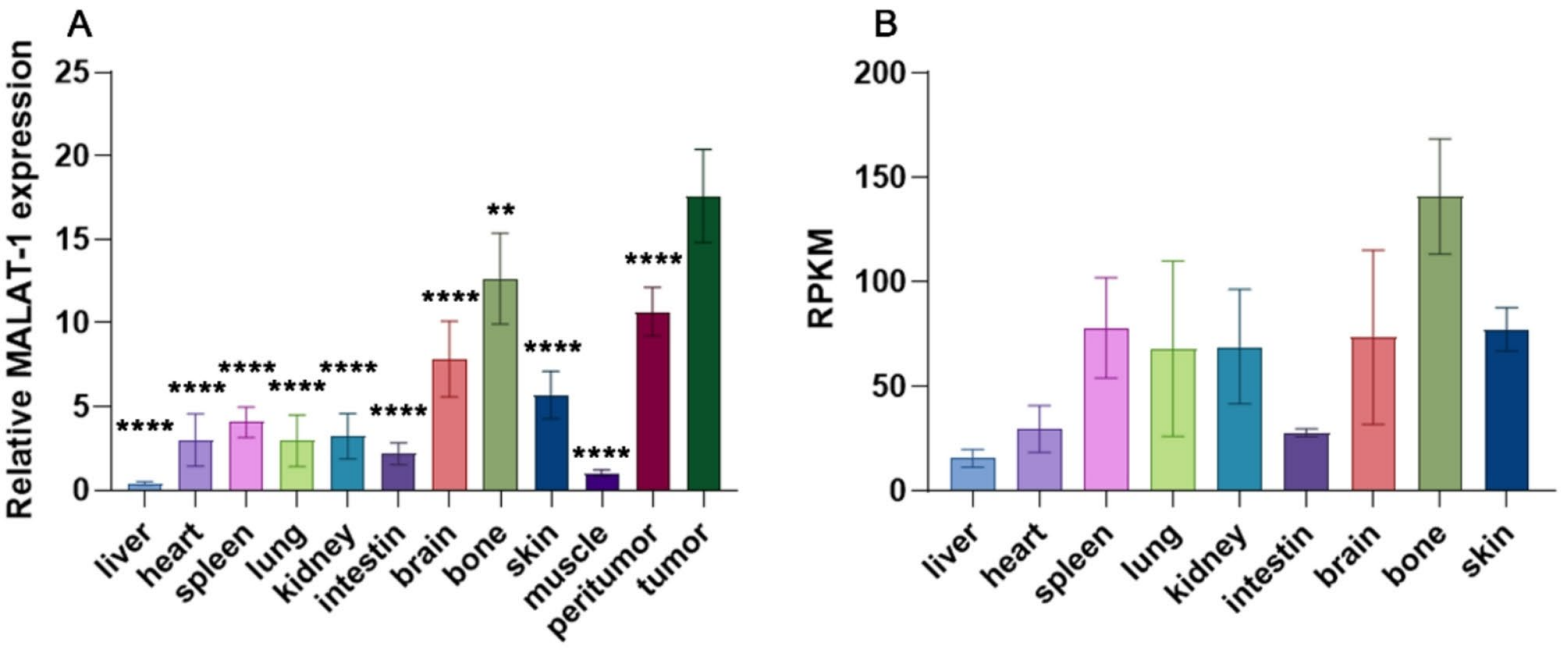
MALAT1 Expression in Nude Mice and Human. (**A**) In nude mice, differences in MALAT1 expression between tumors and various tissues were compared using oneway-ANOVA, **P < 0.01, ****P < 0.001. The level of MALAT1 in muscle tissues was considered as 1. (**B**) in human, cited from NIH BioProject: PRJEB4337. RPKM indicates Reads Per Kilobase per Million mapped reads

We conducted a comparative analysis of MALAT1 expression in our animal model and various organs of the human body. Due to the previously identified lower expression of MALAT1 in A431 (Fig. 1), we selected the A431 xenograft model for our investigation. Figure 4A illustrates the relative expression levels of MALAT1 in different tissues of the A431 xenograft model. MALAT1 expression in the tumor was nearly 18 times higher compared to muscle tissue. Significant differences were observed in the MALAT1 expression of the A431 tumor when compared to peritumor tissue (*P* < 0.001) and other normal tissues (*P* < 0.001, bone *P* < 0.01). Additionally, we referred to the transcriptional analysis database of various human tissues by Fagerberg et al. [33] and selected the MALAT1 gene for analysis, resulting in Fig. 4B. Our analysis suggested relatively higher MALAT1 expression in bone compared to other tissues, with liver displaying the lowest MALAT1 levels. Similar expression trends were observed in both nude mice and human samples (Fig. 4).

### In vivo near-infrared fluorescence imaging

**Fig. 5:**
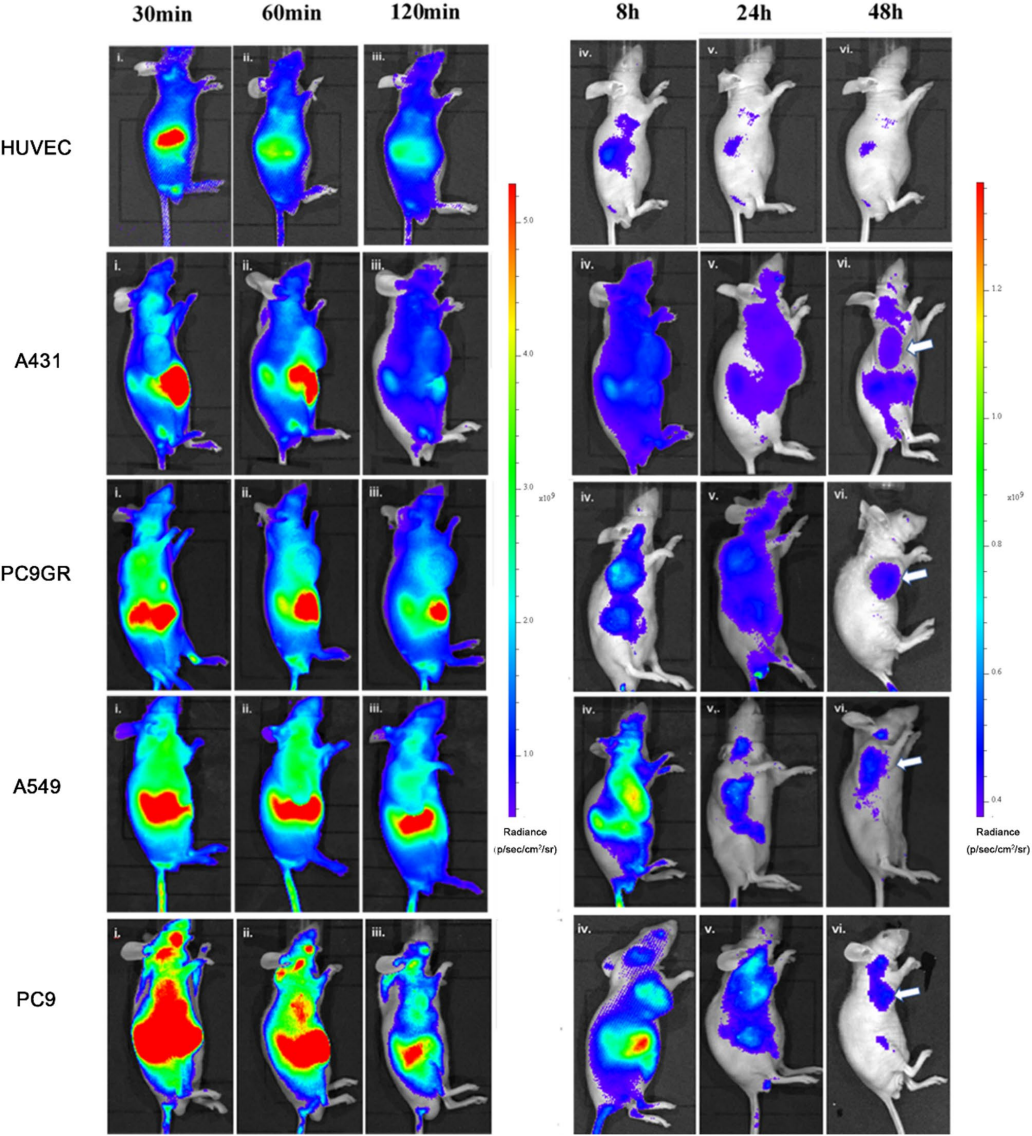
In vivo near-infrared fluorescence imaging of different tumor xenograft models. In vivo near-infrared fluorescence imaging analysis of different tumor xenograft models. Sample size is 6 in each group with 6 images at (i) 30 min, (ii) 60 min, (iii) 120 min, (iv) 8 h, (v) 24 h, and (vi) 48 h. The increasing average radiance is illustrated from blue (low) to red (high), with the 30 min, 60 min, and 120 min images using the same scale on the left, and the 8 h, 24 h, and 48 h images using the same scale on the right. Arrow indicates the area of tumor at 48 h. The probe was administered via tail vein injection

**Fig. 6:**
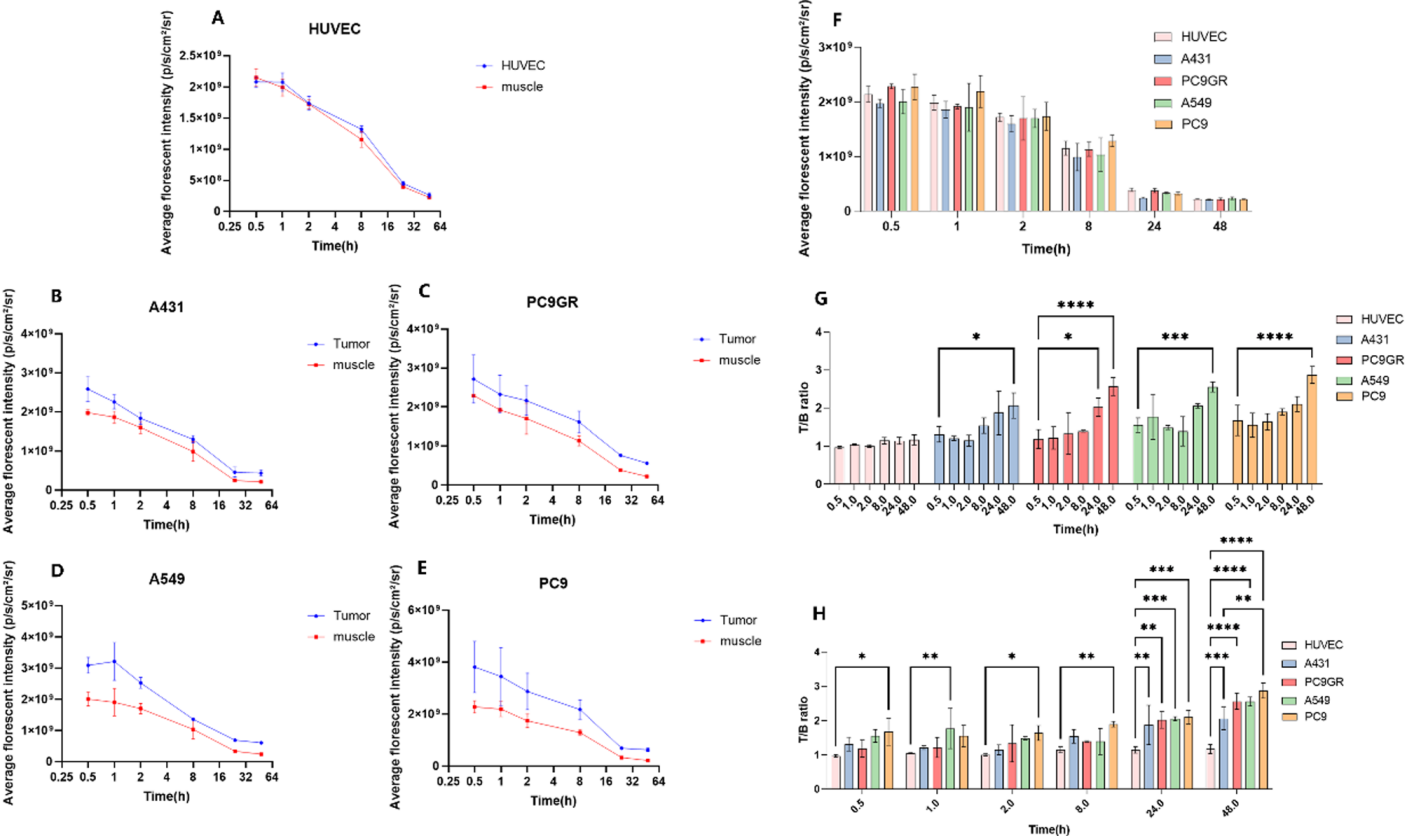
In vivo near-infrared fluorescence imaging analysis. (**A**-**E**) Time-dependent changes of average fluorescence intensity in tumor regions and hind limb muscles. Fluorescence intensity is measured in photons per second per centimeter squared per steradian (p/s/cm^2^/sr), with *N* = 6 in each group. HUVEC cell injection does not induce tumor formation and serves as the control group, the Average Fluorescence Intensity were taken from the injection site. (**F**) Baseline fluorescence intensity in hind limb muscles. All groups received a tail vein injection of 1.5 nmol of Cy5.5-MALAT1 ASO at time 0, with nude mice weighing 20 ± 2 g. (**G**) Tumor/ background ratio (T/B ratio) comparison between different time points. (**H**) Tumor/ background ratio (T/B ratio) comparison between tumors. The fluorescence intensity of tumor tissues were contrasted to the average florescent intensities of the hind limbs in each mouse, The comparison was conducted using student’s t test, with significance levels indicated as *P < 0.05, ***P < 0.005, ****P < 0.001

In vivo fluorescence images of Cy5.5-MALAT1-ASO were obtained and analyzed in the xenograft models. In Fig. 5, HUVEC cells were subcutaneous injected to establish an experimental control, the fluorescence signal at the injection site remained comparable to the surrounding tissues within 48 h. Both tumor and normal tissues displayed the highest fluorescent intensity at 30 min, with the renal region exhibiting the maximum intensity, followed by the tumor and abdomen. However, at 48 h, tumor exhibited the highest florescent intensity compared to normal tissues. Additionally, an independent extended study using the A431 tumor model demonstrated that the fluorescence intensity in the tumor region at 72 h remained more than threefold higher than at 0 h (Figure S6, Table S1), further supporting the long-term stability and tumor-targeting ability of the probe. Figure 6 A shows that the fluorescence intensity at the injection site in HUVEC cells is comparable to that in the hind limb muscles, while in Fig. 6B-E, various tumors exhibited higher fluorescence intensity than hind limbs muscles at different time points. Figure 6 F demonstrates that the hind limb muscles fluorescence intensity did not significantly differ among the groups at the same time. Therefore, hind limbs were considered as background tissue in the groups. Figure 6G-H employed the analysis of the Tumor/Background ratio (T/B ratio). Figure 6G revealed minimal changes in the HUVEC group with T/B ratio around 1, while all tumors showed a significant increase in T/B ratio at 48 h compared to the T/B ratio at 0.5 h. Moreover, the average T/B ratio for all tumor models at 24 h exceeded 2 folds. Figure 6 H showed a significant difference in T/B ratio between all tumor models and the HUVEC model after 24 h. At 48 h, there was a significant difference in T/B ratio between PC9 and A431.

### Ex vivo near-infrared fluorescence imaging

**Fig. 7:**
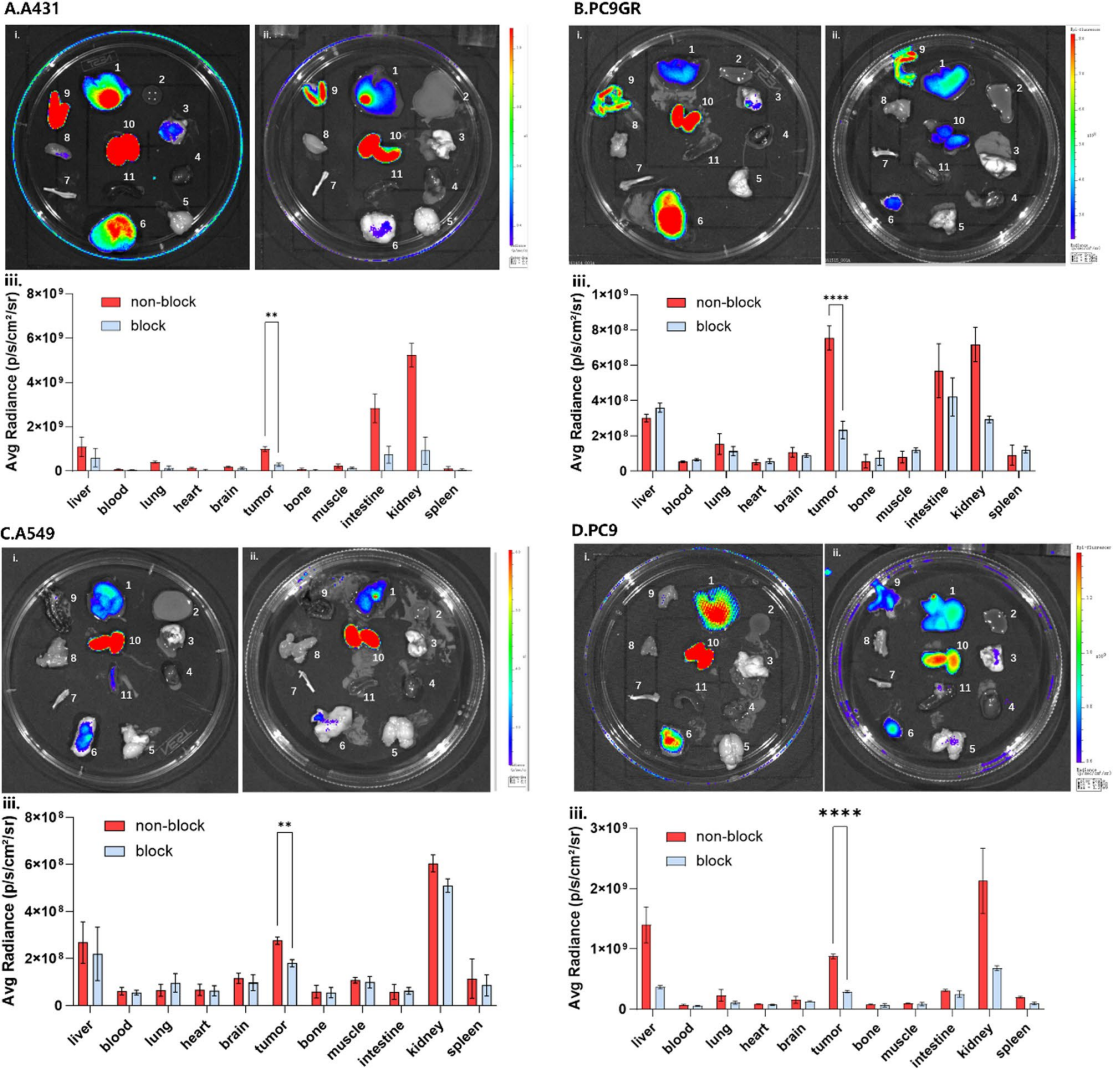
Ex Vivo Near-Infrared Fluorescence Imaging. Organs were harvested 48 h post-injection of the probe. (**A**)A431, (**B**)PC9GR, (**C**) A549, (**D**) PC9. (i) The non-block group (ii) block group (iii) average florescent intensity. Organ display in an order: (1) Liver, (2) Blood, (3) Lung, (4) Heart, (5) Brain, (6) Tumor, (7) Bone, (8) Muscle, (9) Intestine, (10) Kidney, 11. Spleen. Sample size *N* = 6 in non-block group and *N* = 4 in block group. **P < 0.01, ****P < 0.001, two-tailed Student’s t-test

Figure S7 demonstrated the probe distribution in various tissues and organs within HUVEC injected nude mouse (Control group) at different time point. The probe rapidly accumulated in the kidney and liver, and there was also a certain fluorescence signal in the intestine. In Fig. 7, we performed ex vivo imaging and block experiments in various tumor models. Cy5.5-MALAT1-ASO was predominantly taken by tumors at 48 h post injection and the addition of unlabeled MALAT1 ASO for blocking resulted in a significant decrease in fluorescence intensity in tumor tissues.

### Examination of possible liver and kidney damage

To evaluate the potential toxicity of Cy5.5-MALAT1-ASO in vivo, serum biochemical analyses were performed 72 h after injection. Serum levels of alanine aminotransferase (ALT), aspartate aminotransferase (AST), blood urea nitrogen (BUN), and creatinine (CRE) remained within normal ranges and exhibited no significant differences between the two groups. (**Table S2**).

## Discussion

MALAT1, as a highly expressed lncRNA in various tumor tissues, holds the potential to serve as a diagnostic and therapeutic target in cancer. The diagnostic significance of MALAT1 has been confirmed in multiple types of tumors, including lung, liver [34]cervix [35]breast [36]colorectal [37]prostate [38]gastric [39]ovary [40]gallbladder [41]pancreas [42]bone [43] and other cancers such as glioma [44] and multiple myeloma [45]. MALAT1 is also being actively studied as a potential therapeutic target. Xu et al. demonstrated that inhibiting MALAT1 through siRNA targeting significantly reduced the growth, migration, and proliferation of colorectal cancer cells [46]. Arun et al. also showed that using MALAT1-specific antisense oligonucleotides (ASO) to knock down the MALAT1 gene in a mouse breast cancer model resulted in slower primary tumor growth, increased differentiation, and reduced metastasis [47]. Our database analysis reveals elevated MALAT1 expression across various tumors and survival analysis indicates that high MALAT1 expression correlates with an unfavorable prognosis in some tumors, emphasizing the crucial significance of MALAT1 as a target in tumor diagnostic assessments. However, most studies currently focus on diagnosing MALAT1 from body fluids, and there is ongoing debate regarding the sensitivity and specificity of this method. Besides, a challenge in the development of MALAT1-related treatment research is the difficulty in assessing the correlation between therapeutic efficacy and MALAT1 expression levels. There is a lack of a non-invasive, long-term clinical monitoring method for MALAT1 expression.

In this study, our objective was to validate the effectiveness of the Cy5.5-labeled MALAT1-ASO for optical imaging (OI) in the targeted visualization of tumors. This technique allows for non-invasive and precise detection of the expression of MALAT1, offering the dual advantages of high sensitivity and selectivity. Additionally, its real-time monitoring capability provides the potential for studying dynamic changes in MALAT1. Compared to our initial study conducted in liver cancer cells, we have further expanded the application of this probe to lung cancer and skin squamous cell carcinoma [29, 30]. Additionally, we have further investigated the ability of this probe to distinguish tumors with different levels of MALAT1 expression.

We conducted research using lung cancer and skin squamous cell carcinoma cell lines. The research revealed a significant upregulation of MALAT1 expression in A431, PC9GR, A549, and PC9 tumor cell lines relative to the HUVEC cell line, with PC9 exhibiting the highest expression and A431 the lowest. Furthermore, the targeting binding characteristics of the probe were investigated through in vitro cell fluorescence imaging and flow cytometry. We observed specific probe uptake corresponding to MALAT1 expression, with highest fluorescent signal in PC9 and lowest in HUVEC with the same probe concentration. Nearly complete blocking of fluorescent signals was observed after incubation of the unlabeled probe, confirming the probe’s specificity. Additionally, concentration gradient experiments verified the concentration-dependent properties of the probe, and confocal localization analysis revealed a predominantly nuclear fluorescence signal with a minimal cytoplasmic signal, consistent with the intracellular expression pattern of MALAT1 [48], which is mainly localized in nuclear speckles.

In our study, the expression levels of MALAT1 in various tissues of nude mice were further analyzed. Elevated MALAT1 expression was notably observed in tumor tissues when compared to peritumor and normal tissues. Additionally, within normal tissues, a significant upregulation of MALAT1 was noted in bone, with the lowest expression observed in the liver and relatively moderate expression levels in organs such as the kidneys. By comparing human sample databases, we found that the expression distribution of MALAT1 is consistent between nude mouse and human organs.

The Cy5.5-MALAT1 ASO probe was further evaluated in the xenograft model. In comparison to the HUVEC control group, all four types of tumors exhibited rapid probe uptake at the tumor sites. Moreover, due to the stronger binding of the probe to tumors compared to normal tissues, the probe washout in tumors is much slower than normal tissues (Fig. 5). The T/B ratio in all tumor group exceeding 2-fold 24 h after probe injection (Fig. 6G). With time extension, there was an improved imaging effect. Interestingly, in in vivo experiments, we observed variations in probe uptake among different tumors. At 48 h, the probe uptake correlated closely with the expression levels of MALAT1 in the tumors (Fig. 6H). Compared to previous studies on LM3^30^, our research marks the first instance where the probe could serve as a tool for distinguishing different types of tumors based on MALAT1 expression levels. On the other hand, it is noteworthy that the T/B ratio of imaging continues to increase over time, indicating that the optimal imaging time has not been determined in this experiment. For probes labeled with Cy5.5, the optimal imaging time varies significantly depending on the conjugate characteristics. For instance, the imaging time for A Chlorotoxin Cy5.5 Bioconjugate showed significant differences within 14 days [49]whereas another HDAC-Targeted Imaging Probe LBH589-Cy5.5 demonstrated optimal imaging within 6 h [50]. From a clinical perspective, shorter injection-to-detection intervals contribute to convenience. To address this, further modifications could be made to improve the efficiency of our probe.

Ex vivo experiments in normal animals found increased fluorescence levels in the kidneys, followed by the liver and intestines. This could explain the high-intensity fluorescence signal observed in the abdominal region of mice in in vivo experiments. Previous results indicated that MALAT1 expression in renal and hepatic-intestinal tissues was not significant (Fig. 4). This indicates that the high uptake of the fluorescence probe is possibly attributed to probe metabolism, suggesting renal excretion as the primary pathway of the probe and hepatoenteric metabolism as the secondary pathway, which is corroborated the probe’s excretion pathway identified in prior study [29]. However, the accumulation of probes related to high metabolism can interfere with tumor imaging. Therefore, there are certain limitations to detecting tumors in abdominal organs such as the kidneys, liver, and intestines. Additionally, we recommend using skeletal muscles as a baseline for fluorescence intensity because they exhibit lower MALAT1 expression. Moreover, the distribution of the probe in skeletal muscles remains relatively stable under different conditions (Fig. 6F). Moreover, we explored the highly specific binding ability of Cy5.5 MALAT1 ASO through competitive inhibitory ex vivo organ experiments. Administering a large dose of unlabeled MALAT1 ASO before the labeled probe prevented its binding to MALAT1, resulting in a significant decrease in fluorescence intensity in the tumor region. This confirmed that Cy5.5-MALAT1 ASO specifically binds to MALAT1 in the tumor in vivo.

Our research presents a novel diagnostic approach for lung cancer and skin squamous cell carcinoma. Although our study is limited to these two types of tumors, we must acknowledge that other tumors with high MALAT1 expression could also benefit from this probe as a diagnostic tool. However, specific sensitivity and detection parameters need to be validated through experimentation. Early detection and diagnosis of tumors can be achieved by evaluating MALAT1 expression levels. The use of our imaging probe may potentially enable non-invasive, specific, and high-contrast visualization of MALAT1 expression. Furthermore, the development of this imaging probe could open up new strategies for tumor therapy, such as combining it with drugs or radioisotopes for targeted treatment of tumor cells. Additionally, the utilization of this probe system provides a new tool for studying the molecular mechanisms of MALAT1 in tumors.

While our research suggests excellent imaging results, our probe has some limitations. Firstly, despite the high imaging accuracy of Cy5.5 and previous research demonstrating the superior penetration of near-infrared (NIR) light compared to visible light [51]we observed that experimental results may be influenced by factors such as imaging angle and the thickness of the animal body wall. Although our xenograft models avoided the issue of body wall thickness, imaging results for in situ tumors may still be affected. Shallow-level tumors such as lung cancer and skin tumors may exhibit good imaging effects, while further research is needed for imaging effect in deep-level tumors such as bone tumors and blood tumors, etc. Secondly, biological samples can produce autofluorescence in the infrared spectrum. This background signal could interfere with the specific detection of Cy5.5-labeled antisense oligonucleotides targeting MALAT1. Minimizing or distinguishing this autofluorescence from the specific signal of interest can be challenging and may require further experiments. Additionally, all fluorescent probes are susceptible to photobleaching. In our study, strict light avoidance measures were implemented in in vitro experiments. However, in in vivo experiments, where light avoidance was not rigorously controlled, the imaging results revealed that the photobleaching effect does not compromise the quality of our probe’s imaging.

## Conclusion

MALAT1 exhibits distinct expression across various tumors, and elevated MALAT1 expression is linked to poor tumor prognosis. Our study validated the target specificity and high tumor-to-normal ratio of Cy5.5-MALAT1-ASO probe in lung cancer and epidermal carcinoma xenograft models. Moreover, this probe facilitates non-invasive detection and differentiation of MALAT1 expression levels in tumors of live animals. These results suggest the potential for the clinical translation of Cy5.5-MALAT1-ASO in detecting different MALAT1 expression in tumors.

## Electronic supplementary material

Below is the link to the electronic supplementary material.

Supplementary Material 1

## Data Availability

No datasets were generated or analysed during the current study.
