# Supplementary material for: Fluorescence imaging of MALAT1 expression using a Cy5.5-labeled antisense oligonucleotide in lung cancer and epidermal carcinoma cells
Source: Cancer Imaging. 2025 Jul 1;25:82. doi: 10.1186/s40644-025-00903-y (PMC12211275; doi:10.1186/s40644-025-00903-y)
Supplement: Supplementary file 1 — Supplementary Material 1 [file 40644_2025_903_MOESM1_ESM.docx]

**Supplementary Materials**

**Fluorescence imaging of MALAT1 expression using a** **Cy5.5-labeled antisense oligonucleotide in** **lung cancer and epidermal carcinoma cells**

Zhenfeng Liu ^1,†,*^, Chengjun Yao^2,†^ Haopeng Ni^2^, Guolin Wang^1^, and Mengjie Dong ^1,^ *

**
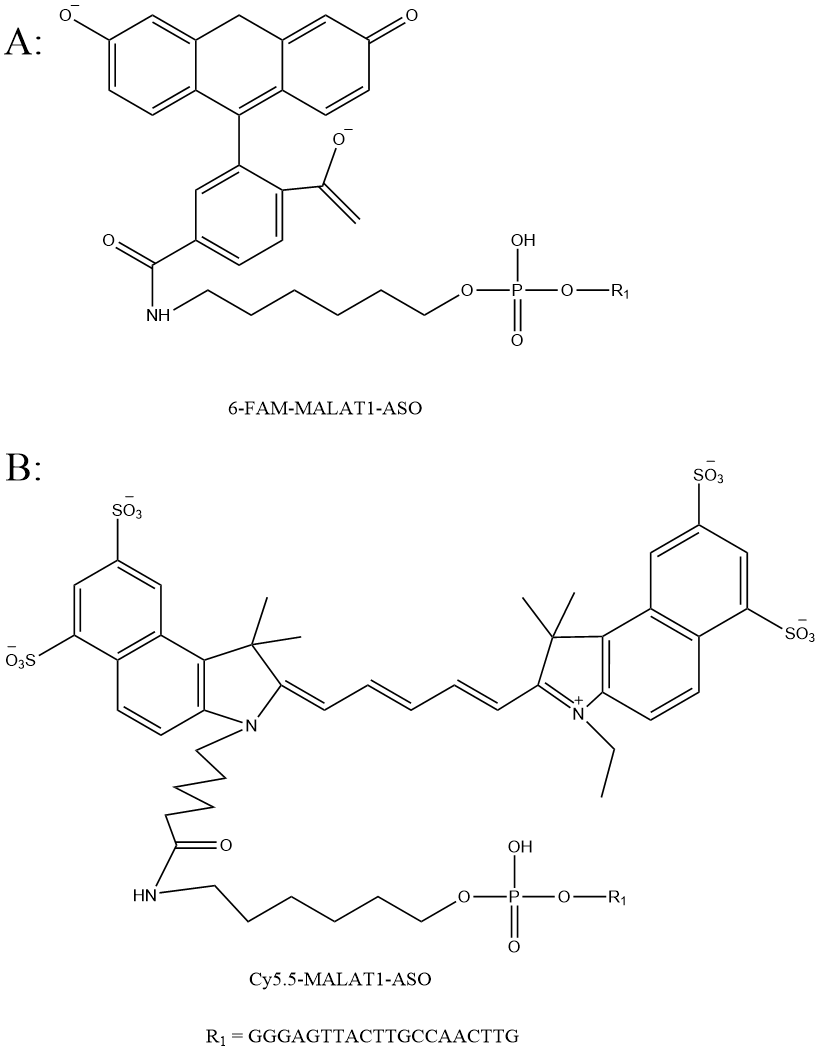
**

**Figure S1. Chemical Structures of Two MALAT1 Probes.** (A) Structure of 6-FAM-labeled MALAT1 antisense oligonucleotide (6-FAM-MALAT1-ASO). (B) Structure of Cy5.5-labeled MALAT1 antisense oligonucleotide (Cy5.5-MALAT1-ASO).


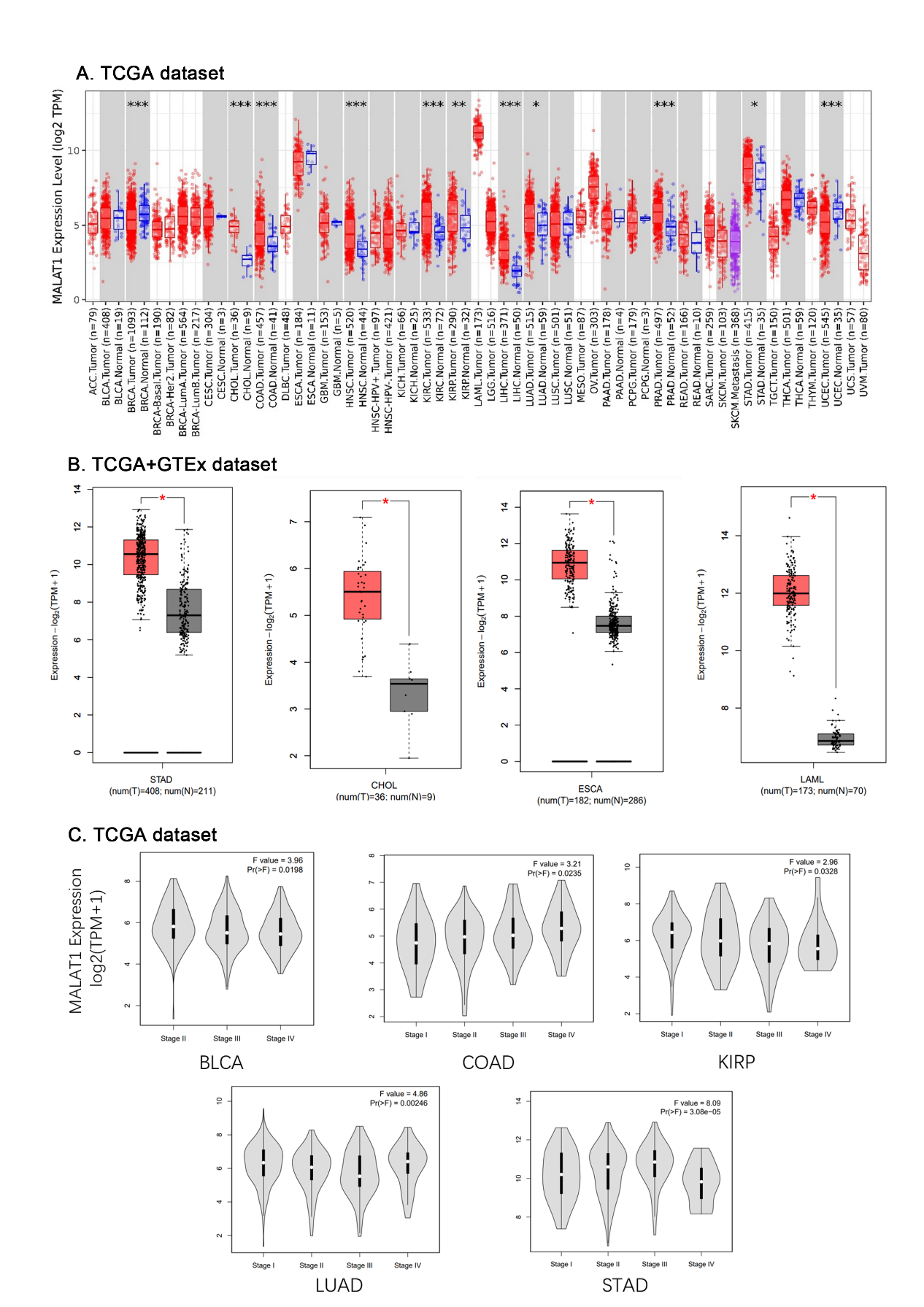


**Figure S2. MALAT1 Expression Alterations Across Various Tumor Types.** (A) MALAT1 expression levels in TCGA cancers were compared with adjacent normal tissues (if available). Significantly elevated MALAT1 expression was observed in Cholangiocarcinoma (CHOL), Colon Adenocarcinoma (COAD), Head and Neck Squamous Cell Carcinoma (HNSC), Kidney Renal Clear Cell Carcinoma (KIRC), Kidney Renal Papillary Cell Carcinoma (KIRP), Liver Hepatocellular Carcinoma (LIHC), Lung Adenocarcinoma (LUAD), Prostate Adenocarcinoma (PRAD), and Stomach Adenocarcinoma (STAD). Conversely, downregulation of MALAT1 was observed in two tumor types, namely Breast Invasive Carcinoma (BRCA) and Uterine Corpus Endometrial Carcinoma (UCEC). (B) Comparison of MALAT1 expression between tumors and normal tissues in the TCGA+GTEx dataset revealed significant MALAT1 upregulation in CHOL, Esophageal Carcinoma (ESCA), Acute Myeloid Leukemia (LAML), and Stomach Adenocarcinoma (STAD) compared to normal tissues. (C) MALAT1 expression differences across tumor stages were examined. Significant differences were observed in Bladder Urothelial Carcinoma (BLCA), COAD, KIRP, LUAD, and STAD. Non-paired Wilcoxon Rank Sum and Signed Rank Tests were employed for differential significance analysis, with statistical significance indicated by P < 0.05.


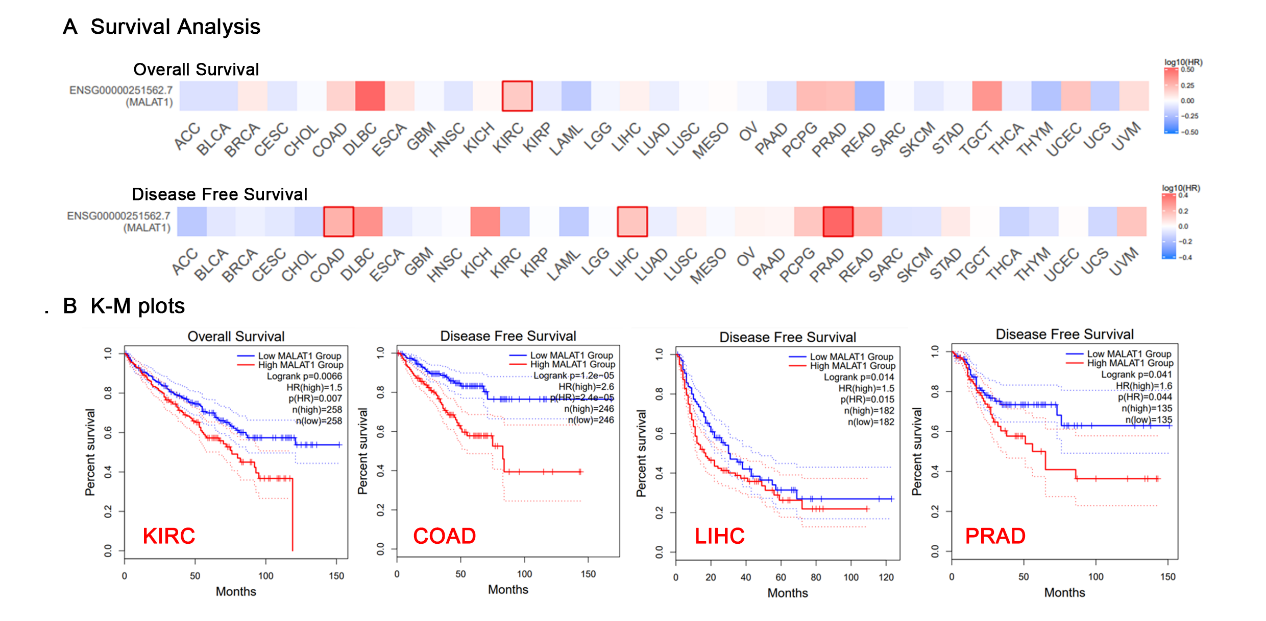


**Figure S3. Pan-cancer MALAT1 Survival Prognosis Analysis**. (A) Overall Survival and Disease Free Survival analysis. The Logrank test method was utilized to assess the significance of differences. (B) Kaplan-Meier plots in tumors with significant difference in survival analysis.


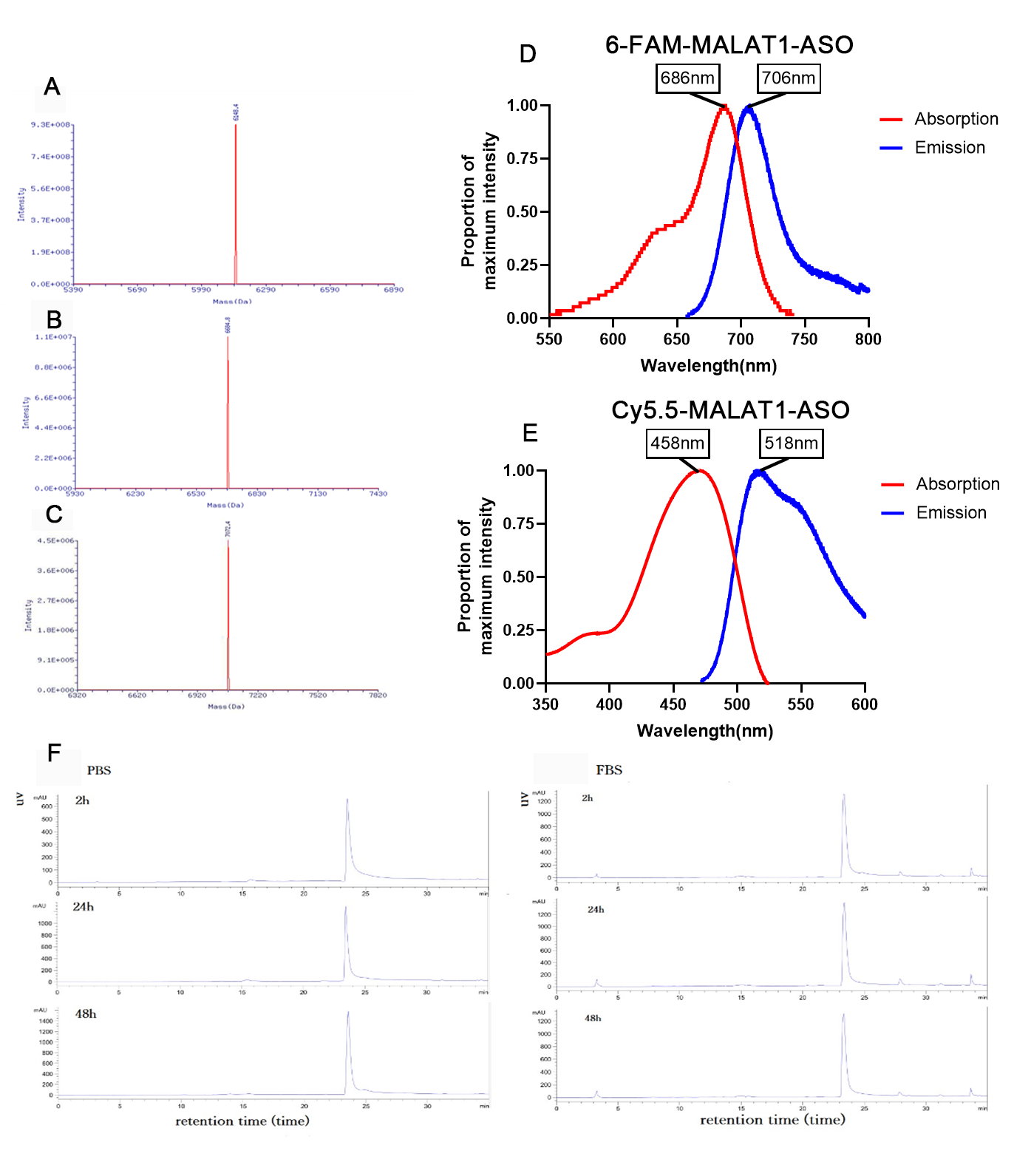


**Figure S4. Physicochemical Characterization of MALAT1-ASO Probes.** (A) Mass spectrometry of MALAT-1-ASO. (B) Mass spectrometry of 6-FAM-MALAT-1-ASO. (C) Mass spectrometry of Cy5.5-MALAT-1-ASO. (D) Absorption and emission spectra of 6-FAM-MALAT-1-ASO. (E) Absorption and emission spectra of Cy5.5-MALAT-1-ASO. (F) In vitro biostability of the Cy5.5-MALAT-1-ASO probe.

**
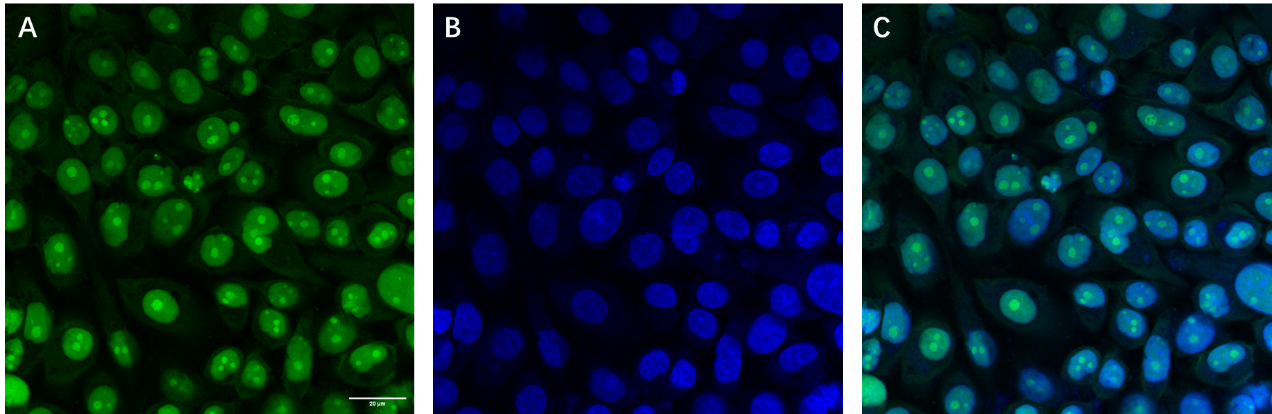
**

**Figure S5. Confocal Fluorescence Supplement for Localization of MALAT1 in PC9 Cells.** (A)6-FAM-MALAT1. (B) DAPI. (C) Merge


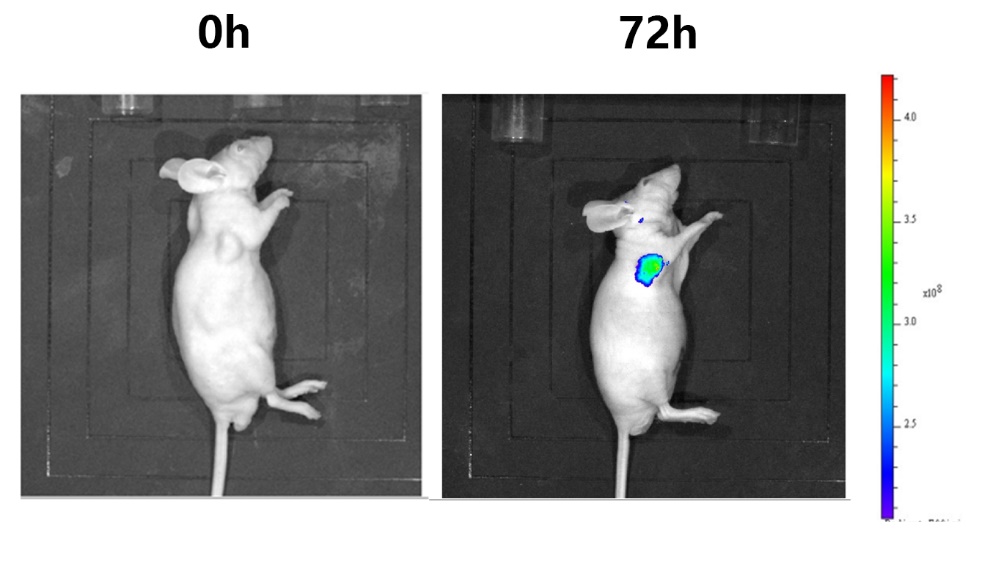


**Figure S6.** **In Vivo Near-Infrared Fluorescence Imaging Following Cy5.5-MALAT1-ASO Injection in A431 Xenograft Models.** Fluorescence imaging was conducted at 0 h and 72 h post-injection in A431 xenograft-bearing nude mice (n = 6). Images were acquired under identical imaging parameters and displayed using the same radiant efficiency scale (p/s/cm²/sr).

**Table S1. Average Radiant Efficiency Between 0h and 72h Post Cy5.5-MALAT1 ASO Injection.**

| Group | 0h | 72h | p |
| --- | --- | --- | --- |
| Avg Radiant Efficiency [p/s/cm2/sr] | 1.1*e8 ± 8.8*e+06 | 3.3*e8 ± 1.2*e+07 | <0.05 |

Note: Data represent mean ± SD. Paired Student’s t-test was used for statistical analysis (n = 6).

**
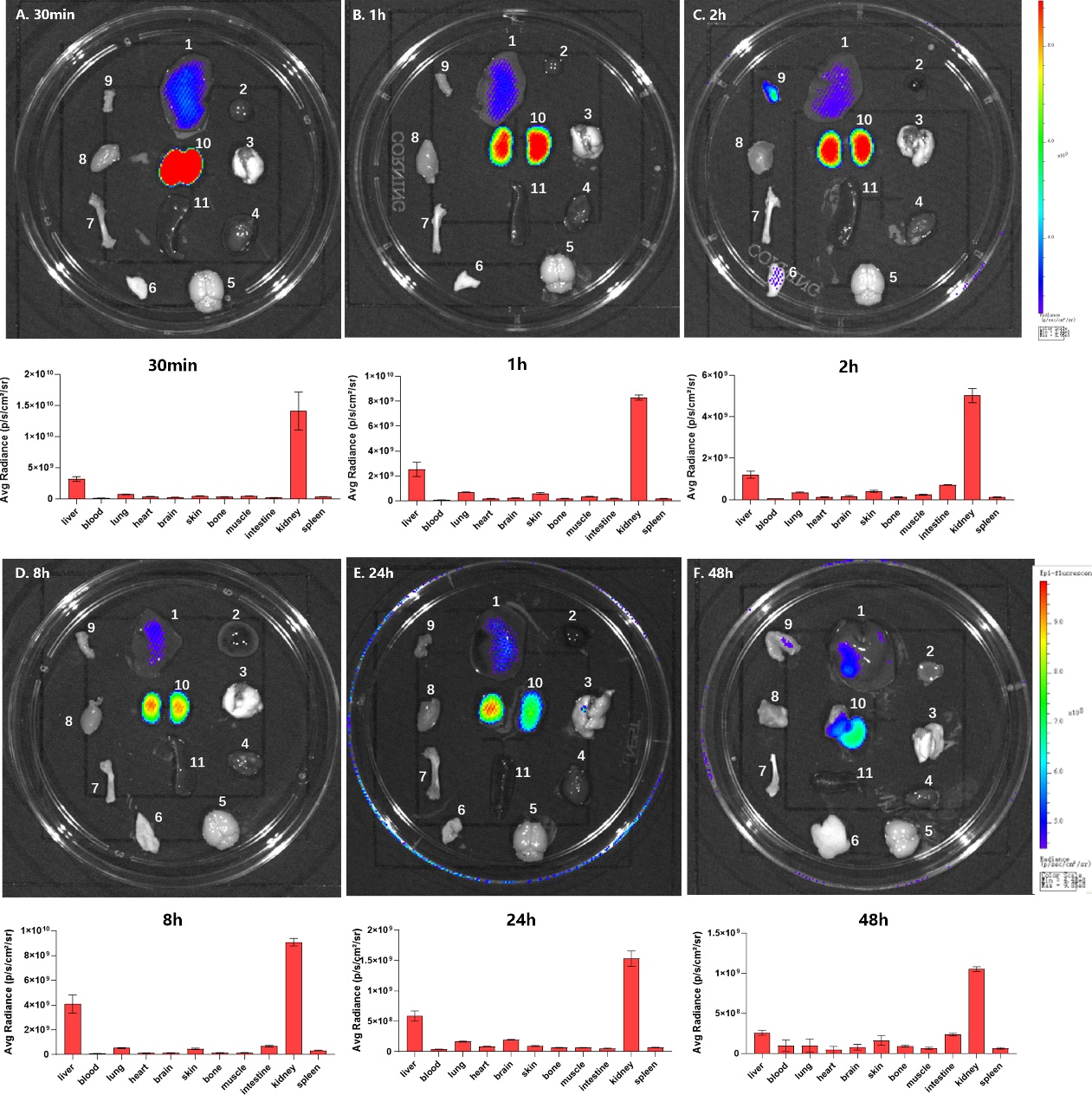
**

**Figure S7. Ex Vivo Near-Infrared Fluorescence Imaging of HUVEC Model at Different Time Point.**

Organs were harvested post-injection of the probe at (A) 30min, (B) 1h, (C)2h, (D) 8h, (E) 24h, (F) 48h. Each figure demonstrates the florescent image and average florescent intensity. Organ display in an order: 1. Liver, 2. Blood, 3. Lung, 4. Heart, 5. Brain, 6. Skin of HUVEC injection site, 7. Bone, 8. Muscle, 9. Intestine, 10. Kidney, 11. Spleen. Sample size N=4.

|  | WBC(10^9/L) | RBC(10^12/L) | HGB(g/L) | MCV(fL) | MCH(pg) | MCHC(g/L) | ALT(u/L) | AST(u/L) | BUN(mmol/L) | CREA(umol/L) | PLT(10^9/L) | HCT(%) |
| --- | --- | --- | --- | --- | --- | --- | --- | --- | --- | --- | --- | --- |
| CTRL | 2.15±0.41 | 8.23±0.50 | 132±30 | 49.8±2.5 | 15.8±2.6 | 315±40 | 77.39±10.21 | 380.75±40.25 | 8.25±0.45 | 6.12±0.45 | 682±250 | 41.2±7.8 |
| Cy5.5-MALAT1-ASO(1.5 nmol) | 2.27±0.39 | 8.53±0.45 | 120±35 | 50.8±3.2 | 15.5±3.2 | 319±57 | 85.39±9.21 | 378.35±50.12 | 8.04±0.65 | 6.35±0.55 | 750±360 | 37.8±8.8 |

**Table S2. Blood Test for Possible Liver and Kidney Damage of Cy5.5-MALAT1-ASO Injection.**

Note: WBC, white blood cells; RBC, red blood cells; HGB, hemoglobin; MCV, mean corpuscular volume; MCH, mean cell hemoglobin; MCHC, mean corpuscular hemoglobin concentration; ALT, alanine aminotransferase; AST, aspartate aminotransferase; BUN, blood urea nitrogen; CREA, creatinine; PLT, platelets; HCT, hematocrit. Blood samples were analyzed using a certified automated hematology analyzer (n = 6).
